# Associations of midpoint of sleep and night sleep duration with type 2 diabetes mellitus in Chinese rural population: the Henan rural cohort study

**DOI:** 10.1186/s12889-021-10833-6

**Published:** 2021-05-07

**Authors:** Zhihan Zhai, Xiaotian Liu, Haiqing Zhang, Xiaokang Dong, Yaling He, Miaomiao Niu, Mingming Pan, Chongjian Wang, Xiaoqiong Wang, Yuqian Li

**Affiliations:** 1 Department of Epidemiology and Biostatistics, College of Public Health, Zhengzhou University, 100 Kexue Avenue, Zhengzhou, 450001 Henan PR China; 2 Department of Preventive Medicine, Henan University of Chinese Medicine, 156 East Jinshui, Zhengzhou, Henan 450046 PR China; 3 Department of Economics, Business School, Zhengzhou University, Zhengzhou, Henan PR China; 4 Department of Clinical Pharmacology, School of Pharmaceutical Science, Zhengzhou University, Zhengzhou, Henan PR China

**Keywords:** Midpoint of sleep, Night sleep duration, Type 2 diabetes mellitus, Rural population

## Abstract

**Background:**

The study aimed to investigate the independent and combined effects of midpoint of sleep and night sleep duration on type 2 diabetes mellitus (T2DM) in areas with limited resources.

**Methods:**

A total of 37,276 participants (14,456 men and 22,820 women) were derived from the Henan Rural Cohort Study. Sleep information was assessed based on the Pittsburgh Sleep Quality Index. Logistic regression models and restricted cubic splines were used to estimate the relationship of the midpoint of sleep and night sleep duration with T2DM.

**Results:**

Of the 37,276 included participants, 3580 subjects suffered from T2DM. The mean midpoint of sleep among the Early, Intermediate and Late groups were 1:05 AM ±23 min, 1:56 AM ±14 min, and 2:57 AM ±34 min, respectively. Compared to the Intermediate group, adjusted odds ratios (*ORs*) and 95% confidence interval (*CI*) of T2DM were 1.13 (1.04–1.22) and 1.14 (1.03–1.26) in the Early group and the Late group. Adjusted *OR* (95% *CI*) for T2DM compared with the reference (7- h) was 1.28 (1.08–1.51) for longer (≥ 10 h) night sleep duration. The combination of late midpoint of sleep and night sleep duration (≥ 9 h) increased 38% (95% *CI* 10–74%) prevalence of T2DM. These associations were more obvious in women than men.

**Conclusions:**

Late and early midpoint of sleep and long night sleep duration were all associated with higher prevalence of T2DM. Meanwhile, midpoint of sleep and night sleep duration might have combined effects on the prevalence of T2DM, which provided potential health implications for T2DM prevention, especially in rural women.

**Trial registration:**

The Henan Rural Cohort Study has been registered at Chinese Clinical Trial Register (Registration number: ChiCTR-OOC-15006699). Date of registration: 2015-07-06.

**Supplementary Information:**

The online version contains supplementary material available at 10.1186/s12889-021-10833-6.

## Background

Type 2 diabetes mellitus (T2DM), a major chronic disease nowadays, has imposed enormous financial pressures on national health care systems and national economies worldwide. It was reported that the number of adults with diabetes worldwide aged 20–79 years reached 463 million, which will increase to 578.4 million by 2030 and 700.2 million by 2045 [1]. In 2019, diabetes was estimated to contribute to one in nine deaths among adults aged 20–79 [2, 3]. As the country with the largest number of T2DM patients, it is urgent to find out the determinants of T2DM to prevent this public health problem in China.

Large prospective studies have demonstrated that long or short night sleep duration was associated with increased risk of T2DM [4–7]. Sleep timing is another important sleep variable that represents a person’s sleep preferences besides night sleep duration. A prospective study from Korea examining the impact of sleep time on the incidence of T2DM showed that habitual late sleep was related to higher risk of T2DM [8]. Chronotype, a trait determining individual circadian preference, is reported to be associated with many lifestyle-related diseases [9–11]. In one analysis of 1620 middle-aged participants from the Korean Genome and Epidemiology Study, evening chronotype was independently associated with diabetes, metabolic syndrome, and sarcopenia [12]. A study of 1014 non-shift working adults in Bangkok with prediabetes reported a significant association between late chronotype and higher HbA1c levels [13]. A recent study from the UK Biobank also showed that late chronotype increased the risk of T2DM [14].

Midpoint of sleep, the midpoint between sleep and wake-up time, strongly correlates with both chronotype and dim light melatonin onset [15, 16]. Epidemiological studies have shown that late midpoint of sleep was associated with dietary intake and dietary behavior [17–19], depression among adolescents [20], individuals’ well-being [21], and a higher risk of gestational diabetes [22]. However, evidence on the relationship between midpoint of sleep and T2DM in areas with limited resources was scarce and no previous studies explored joint associations of midpoint of sleep and night sleep duration with T2DM.

Therefore, using the baseline data of the Henan Rural Cohort Study, the present study was conducted aimed to investigate the independent and combined associations of the midpoint of sleep and night sleep duration with T2DM in rural Chinese adults.

## Methods

### Study population

The present study was conducted using the baseline data of the Henan Rural Cohort Study. Details of the study have been reported elsewhere [23]. Baseline data collection was obtained from July 2015 to September 2017. In total, 39,259 adults aged 18–79 years completed the baseline survey, including questionnaire, anthropometric and laboratory tests. After excluding participants who were shift workers (*N* = 1553), T1DM patients (*N* = 4), had missing information on sleep assessment and T2DM diagnosis (*N* = 99), 37,276 were retained in the present study for analysis.

This research protocol was approved by the Life Science Ethics Committee of Zhengzhou University. All participants signed informed consent.

### Data collection and assessment of covariates

Information on demographics, lifestyle characteristics, and family disease history was collected by a structured questionnaire. According to previous classification criteria [6], marital status was grouped into two groups: married or cohabitating unmarried and unmarried, divorced or widowed. Education levels were divided into primary school or below, junior high school, and senior high school or above. The average monthly income was categorized into less than 500, 500-, and 1000 or above. Adequate vegetables and fruit intake (> 500 g/day consumption of vegetables and fruit) and high-fat diet (> 75 g/day consumption of meat from livestock and poultry) were defined separately. Smoking and alcohol drinking were grouped into never, former, and current. International Physical Activity Questionnaire (IPAQ) [24] was used to evaluated physical activity (light, moderate, and vigorous). Anthropometric tests were conducted by trained staff according to a standard guideline. Body mass index (BMI) was calculated from the weight and height obtained.

### Sleep variables

Sleep variables (sleep timing, sleep latency snoring, and so on) were obtained by a face-to-face interview based on the Pittsburgh Sleep Quality Index (PSQI). The PSQI consists of 19 items, divided into 7 components, each with a score of 0–3 and a total score of 21, which is used to assess sleep quality [25]. In the present study, the subjects reported bedtime and wake-up time (the time they usually went to bed and got up) and sleep latency (the minutes it took to fall asleep) in the past month. Using these data, sleep time was computed as bedtime plus sleep latency. The midpoint of sleep was calculated as the halfway point between sleep time and wake-up time and was classified into three categories by the 25th and 75th percentile according to the distribution: Early, Intermediate (reference), and Late [26]. Sleep duration was also calculated from sleep time and wake-up time and grouped as < 5 h, 5- h, 6- h, 7- h (reference), 8- h, 9- h, and ≥ 10 h [27]. In addition, information on napping frequency and napping duration were also obtained.

### Definition of T2DM

Subjects were defined as T2DM passing the following criteria: (1) the fasting blood glucose ≥ 7.0 mmol/L or glycated hemoglobin ≥ 6.5%; (2) self-reported being diagnosed with T2DM by a physician or taking hypoglycemic drugs in the past 2 weeks.

### Statistical analyses

Continuous variables are presented as the mean ± SD and categorical variables are presented as the mean ± SD. The t-test (continuous variables) and chi-square test (categorical variables) were used for comparing baseline characteristics of the participants among different T2DM groups. We performed a multiplicative interaction term between gender and the midpoint of sleep or night sleep duration in the regression models and found potential gender interactions (*P* < 0.05). Hence the results were presented by gender (men and women). Moreover, to explore the associations of the midpoint of sleep and night sleep duration with T2DM, logistic regression models were performed by controlling for age, gender, physical activity, marital status, smoking status, drinking status, educational level, average monthly income, BMI, high fat diet, adequate vegetable and fruit intake, napping duration, family history of diabetes, sleep quality, snoring, midpoint of sleep or night sleep duration. The dose-response associations between the midpoint of sleep and night sleep duration with T2DM were evaluated by the three-knots restricted cubic spline. The three knots were 25th, 50th, and 95th percentiles for the midpoint of sleep and 15th, 50th, and 85th percentiles for the night sleep duration according to the distribution. Besides, stratified analyses were performed to assess the potential interaction. Furthermore, night sleep duration was regrouped into < 6 h, 6- h, 7- h, 8- h, ≥ 9 h to investigate the combined effects of the midpoint of sleep and night sleep duration on T2DM by taking the Intermediate group and 7- h of night sleep duration as the reference. Data were analyzed by using the SAS 9.1 (SAS Institute) and R Language software (version 4.0.2). Statistical significance was based on two-tailed *P* < 0.05.

## Results

### Characteristics of participants

Comparisons of characteristics according to T2DM status are presented in Table 1. Of the 37,276 participants, 3580 subjects suffered from T2DM. Compared with those without T2DM, individuals with T2DM were older, and more likely to be women, unmarried, and had lower education level, lower average monthly income, less physical activity, higher BMI, higher PSQI scores and a family history of T2DM. Meanwhile, they tend to have long night sleep and napping durations and early midpoint of sleep. The study participants according to midpoint of sleep showed significant differences for baseline characteristics. The mean midpoint of sleep among the 3 categories were1:05 AM ±23 min, 1:56 AM ±14 min, and 2:57 AM ±34 min, respectively. Details of the baseline characteristics of participants are provided in Supplementary Table 1.

**Table 1:** The demographic characteristics of subjects according to T2DM by gender

Variables	Total			Men			Women		
	Non-T2DM	T2DM	*P*	Non-T2DM	T2DM	*P*	Non-T2DM	T2DM	*P*
N	33,696	3580		13,128	1328		20,568	2252	
Age(year), mean ± SD	55.51 ± 12.23	60.58 ± 9.12	< 0.001	56.92 ± 12.29	59.74 ± 9.7	< 0.001	54.61 ± 12.11	61.08 ± 8.73	< 0.001
Marital status, n (%)			0.018			0.011			< 0.001
Married/cohabitation	30,210 (89.65)	3164 (88.38)		11,747 (89.48)	1218 (91.72)		18,463 (89.77)	1946 (86.41)	
Unmarried/divorced/widowed	3486 (10.35)	416 (11.62)		1381 (10.52)	110 (8.28)		2105 (10.23)	306 (13.59)	
Educational levels, n (%)			< 0.001			0.561			< 0.001
Primary school or below	15,129 (44.90)	2013 (56.23)		4573 (34.83)	459 (34.56)		10,556 (51.32)	1554 (69.01)	
Junior high school	13,498 (40.06)	1161 (32.43)		6063 (46.18)	601 (45.26)		7435 (36.15)	560 (24.87)	
Senior high school or above	5069 (15.04)	406 (11.34)		2492 (18.98)	268 (20.18)		2577 (12.53)	138 (6.13)	
Average income per month, n (%)			< 0.001			0.535			< 0.001
< 500 RMB	12,125 (35.98)	1430 (39.94)		4880 (37.17)	509 (38.33)		7245 (35.22)	921 (40.90)	
500- RMB	11,147 (33.08)	1154 (32.23)		4176 (31.81)	426 (32.08)		6971 (33.89)	728 (32.33)	
≥ 1000 RMB	10,424 (30.94)	996 (27.82)		4072 (31.02)	393 (29.59)		6352 (30.88)	603 (26.78)	
High vegetables and fruits intake, n (%)	14,363 (42.63)	1271 (35.51)	< 0.001	5732 (43.67)	506 (38.13)	< 0.001	8631 (41.96)	765 (33.97)	< 0.001
High fat diet, n (%)	6482 (19.24)	573 (16.01)	< 0.001	3291 (25.07)	310 (23.34)	0.166	3191 (15.51)	263 (11.68)	< 0.001
Smoker, n (%)	6428 (19.08)	499 (13.94)	< 0.001	6375 (48.56)	491 (36.97)	< 0.001	53 (0.26)	8 (0.36)	0.61
Drinker, n (%)	6033 (17.90)	514 (14.36)	< 0.001	5470 (41.67)	484 (36.45)	< 0.001	563 (2.74)	30 (1.33)	< 0.001
Physical activity, n (%)			< 0.001			< 0.001			< 0.001
Light	10,545 (31.29)	1414 (39.50)		4564 (34.77)	580 (43.67)		5981 (29.08)	834 (37.03)	
Moderate	12,823 (38.05)	1261 (35.22)		3618 (27.56)	357 (26.88)		9205 (44.75)	904 (40.14)	
Vigorous	10,328 (30.65)	905 (25.28)		4946 (37.68)	391 (29.44)		5382 (26.17)	514 (22.82)	
Family history of diabetes, n (%)	1181 (3.50)	346 (9.66)	< 0.001	374 (2.85)	133 (10.02)	< 0.001	807 (3.92)	213 (9.46)	< 0.001
Body mass index	24.68 ± 3.53	26.15 ± 3.68	< 0.001	24.35 ± 3.43	26 ± 3.54	< 0.001	24.89 ± 3.57	26.25 ± 3.76	< 0.001
Night sleep duration, mean ± SD	7.75 ± 1.27	7.87 ± 1.36	< 0.001	7.74 ± 1.28	7.88 ± 1.24	< 0.001	7.76 ± 1.26	7.87 ± 1.42	< 0.001
PSQI scores, mean ± SD	3.77 ± 2.71	4.01 ± 2.88	< 0.001	3.30 ± 2.36	3.31 ± 2.20	0.951	4.08 ± 2.88	4.45 ± 3.15	< 0.001
Snoring, n (%)	10,693 (41.8)	1239 (49.2)	< 0.001	5271 (51.3)	527 (55.1)	0.027	5422 (35.4)	712 (45.6)	< 0.001
Napping duration, mean ± SD	57.06 ± 50.38	61.00 ± 50.5	< 0.001	62.85 ± 49.91	65.4 ± 49.17	0.075	53.37 ± 50.33	58.4 ± 51.1	< 0.001
Midpoint of sleep, mean ± SD	1.93 ± 0.79	1.83 ± 0.80	< 0.001	1.92 ± 0.84	1.85 ± 0.85	0.003	1.94 ± 0.75	1.81 ± 0.77	< 0.001

### Comparison of bedtime, wake-up time and sleep duration across 3 categories of the midpoint of sleep

The differences in bedtime, wake-up time and sleep duration (Supplementary Table 2.) across 3 categories of the midpoint of sleep were statistically significant. People with an early midpoint of sleep woke 85 min earlier and went to bed 117 min earlier than people with a late midpoint of sleep. Compared with the Intermediate group (7 h 43 min), the Early group had a longer night sleep duration (8 h 11 min) and the Late group had a shorter night sleep duration (7 h 19 min). In addition, differences were found between men and women.

### Associations of midpoint of sleep and night sleep duration with T2DM

To further explore the associations between midpoint of sleep/night sleep duration and T2DM, logistic regression models were performed for odds ratios (*ORs*) and 95% confidence intervals (*CIs*) (Table 2). Compared with the Intermediate group, people with early midpoint of sleep and late midpoint of sleep had higher odds of T2DM, the *ORs* (95% *CIs*) were 1.13 (1.04–1.22) and 1.14 (1.03–1.26) after adjustment for age, gender, marital status, educational levels, average income per month, high vegetables and fruits intake, high fatty diet, smoking status, drinking status, physical activity, body mass index, family history of diabetes, napping duration, sleep quality, snoring and night sleep duration. Stratification by gender revealed that the association between the midpoint of sleep and T2DM was not significant in both men and women in the adjusted model.

**Table 2:** Odd ratios of T2DM according to midpoint of sleep and night sleep duration by gender

	Total			Men			Women		
	Case/N	Model 1	Model 2	Case/N	Model 1	Model 2	Case/N	Model 1	Model 2
Midpoint of sleep									
Early	1324/11560	1.10 (1.01–1.19)	1.13 (1.04–1.22)	482/4664	1.11 (0.97–1.26)	1.15 (1.00–1.32)	842/6896	1.09 (0.98–1.20)	1.11 (1.00–1.23)
Intermediate	1483/16456	1.00	1.00	540/6220	1.00	1.00	943/10236	1.00	1.00
Late	773/9260	1.14 (1.04–1.25)	1.14 (1.03–1.26)	306/3572	1.16 (0.99–1.35)	1.17 (0.99–1.37)	467/5688	1.09 (0.97–1.23)	1.09 (0.96–1.24)
Night sleep duration									
< 5	71/619	1.16 (0.90–1.50)	1.12 (0.84–1.50)	15/247	0.62 (0.37–1.06)	0.71 (0.40–1.25)	56/372	1.49 (1.10–2.00)	1.51 (1.07–2.14)
5-	137/1662	0.87 (0.72–1.04)	0.84 (0.69–1.04)	44/689	0.68 (0.49–0.94)	0.69 (0.49–0.97)	93/973	0.98 (0.78–1.23)	0.98 (0.76–1.27)
6-	496/5995	0.89 (0.80–1.00)	0.88 (0.78–0.98)	199/2482	0.87 (0.73–1.04)	0.87 (0.73–1.04)	297/3513	0.09 (0.78–1.04)	0.88 (0.76–1.02)
7-	1144/12505	1.00	1.00	456/4979	1.00	1.00	688/7526	1.00	1.00
8-	1027/10621	1.02 (0.93–1.11)	1.02 (0.93–1.11)	375/3861	1.03 (0.89–1.19)	1.02 (0.88–1.19)	652/6760	1.02 (0.91–1.15)	1.02 (0.91–1.15)
9-	491/4373	1.10 (0.98–1.23)	1.11 (0.99–1.25)	169/1621	1.06 (0.88–1.28)	1.08 (0.89–1.31)	322/2752	1.13 (0.98–1.31)	1.14 (0.98–1.32)
≥ 10	214/1501	1.32 (1.13–1.55)	1.28 (1.08–1.51)	70/577	1.22 (0.93–1.60)	1.23 (0.93–1.64)	144/924	1.37 (1.12–1.68)	1.31 (1.07–1.61)

Model 1: adjusted for age, gender (only for total participants);

Model 2: adjusted for age, gender (only for total participants), marital status, educational levels, average income per month, high vegetables and fruits intake, high fatty diet, smoking status, drinking status, physical activity, body mass index, family history of diabetes, napping duration, sleep quality, snoring, night sleep duration or midpoint of sleep

Compared with individuals who slept 7- h, longer (≥ 10 h) night sleep duration was relevant to higher odds of T2DM in the adjusted model, the *OR* was 1.28 (1.08–1.51). Gender stratification showed that the association was more pronounced in women but not statistically significant in men. The *ORs* and 95%*CI* were 1.51 (1.07–2.14) and 1.31 (1.07–1.61) for women who slept < 5 h and ≥ 10 h per night.

**Fig. 1:**
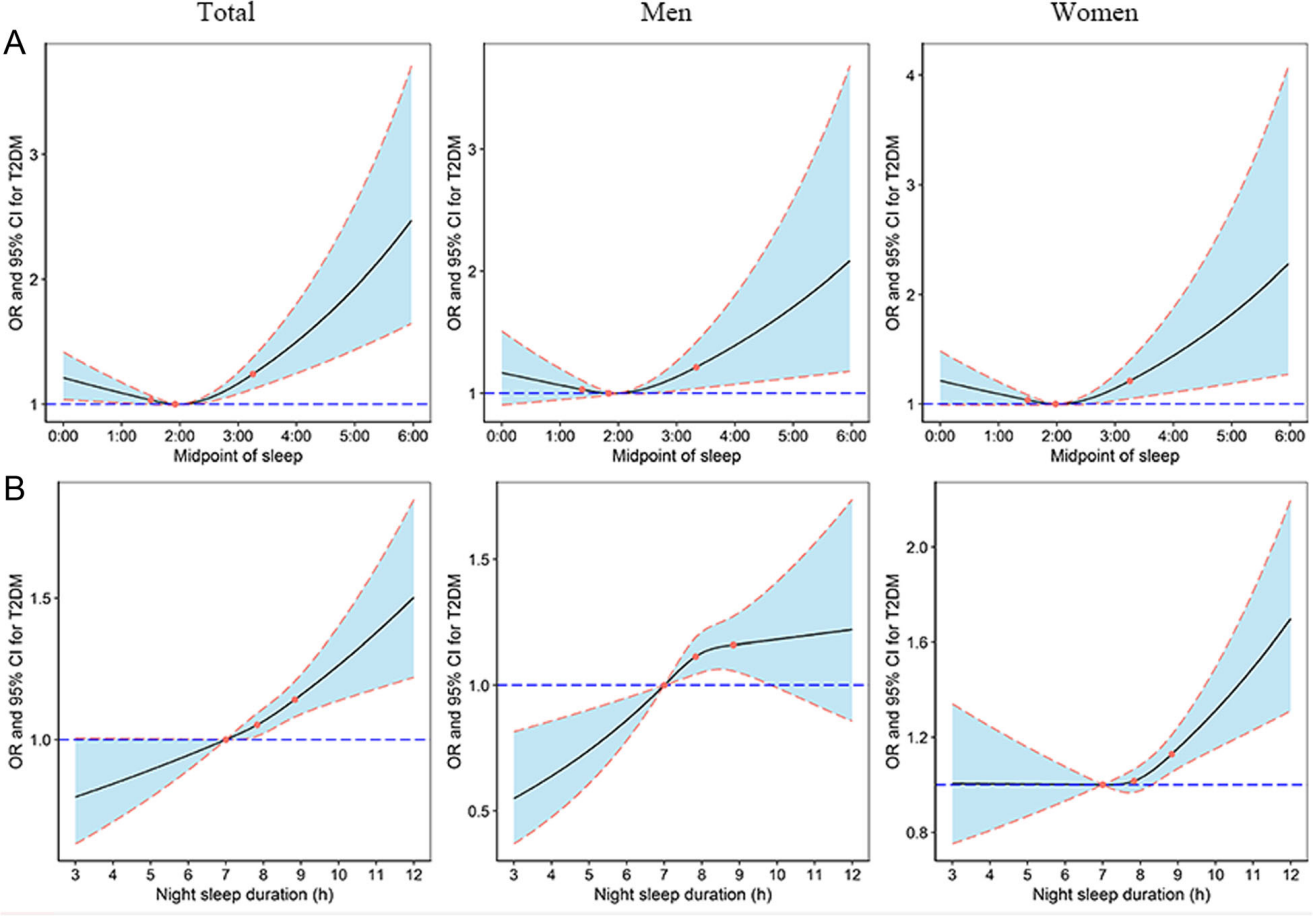
Associations of midpoint of sleep (**a**) and night sleep duration (**b**) with T2DM. Adjusted for age, gender (only for total participants), marital status, educational levels, average income per month, high vegetables and fruits intake, high fatty diet, smoking status, drinking status, physical activity, body mass index, family history of diabetes, napping duration, sleep quality, snoring, night sleep duration or midpoint of sleep

Furthermore, potential associations between midpoint of sleep/night sleep duration and T2DM were also assessed by restricted cubic spline (Fig. 1). A U-shaped dose-response relationship between the midpoint of sleep and T2DM was found after controlling for potential confounders (*P* for nonlinearity < 0.001). Gender stratification showed that the U-shaped dose-response association was significant in both men (*P* for nonlinearity =0.0194) and women (*P* for nonlinearity =0.0036). As for night sleep duration, the U-shaped dose-response relationship was only found in women (*P* for nonlinearity =0.0302). In total participants and men, restricted cubic spline showed that *P* for overall association < 0.05 and *P* for nonlinearity > 0.05 (*P* for nonlinearity =0.5338 for total participants and *P* for nonlinearity =0.1068 for men).

### Subgroup analysis of the association of midpoint of sleep and night sleep duration with T2DM

The results of the associations between midpoint of sleep/night sleep duration and T2DM stratified by age, BMI, family history of diabetes, physical activity, smoking and drinking status are presented in Supplementary Table 3. The effects of the midpoint of sleep on T2DM were significantly modified by age. Participants less than 60 years had statistically stronger associations than the others with early midpoint of sleep (*OR* 1.32, 95%*CI* 1.15–1.52 vs. *OR* 1.11, 95%*CI* 1.00–1.23), but participants ≥ 60 years had statistically stronger associations than the others with late midpoint of sleep (*OR* 1.20, 95%*CI* 1.04–1.39 vs. *OR* 0.92, 95%*CI* 0.80–1.05). Furthermore, current smoking and past drinking had stronger associations with T2DM in both the Early and Late groups.

### Combined effects of midpoint of sleep and night sleep duration on T2DM

**Fig. 2:**
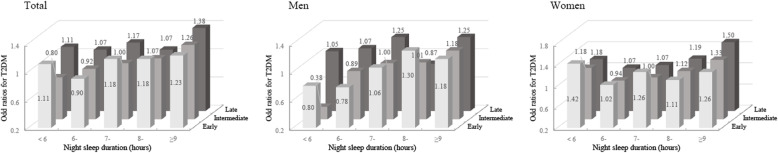
Joint effects of midpoint of sleep and night sleep duration on T2DM. Adjusted for age, gender (only for total participants), marital status, educational levels, average income per month, high vegetables and fruits intake, high fatty diet, smoking status, drinking status, physical activity, body mass index, family history of diabetes, napping duration, sleep quality and snoring

Figure 2 displays a combined analysis of the midpoint of sleep and night sleep duration with T2DM. Compared with those with intermediate midpoint of sleep and 7- h of night sleep duration, individuals with a combined late midpoint of sleep and longer night sleep duration (*OR* 1.38, 95%*CI* 1.10–1.74) had the highest odds of T2DM. Gender stratification showed that this combined association was enhanced in women, which showed a notably U relationship with T2DM.

## Discussion

This study provided new evidence between the midpoint of sleep and T2DM in rural populations, which had implications for a better understanding of the relationship between chronotype and T2DM. A U-shaped dose-response relationship between the midpoint of sleep and T2DM was found in the present study, which indicates that early and late midpoint of sleep may increase the higher odds of T2DM. Besides, late midpoint of sleep and longer night sleep duration might have combined effects on the prevalence of T2DM. Moreover, gender and age might influence the health effects of sleep which should be taken into consideration when making strategies to improve public health in rural areas.

Consistent with previous studies, we found late midpoint of sleep was related to increased odds of T2DM. For instance, former studies showed that late chronotype assessed by chronotype questionnaire was associated with increased risk of T2DM [28]. Moreover, chronotype acquired by free-day mid-sleep time also showed similar results. In a community-based study among Hispanic/Latino adults aged 18–74 years, later sleep timing based on the midpoint of sleep had increased estimated insulin resistance (HOMA-IR) [29]. Furthermore, late chronotype derived from midpoint of sleep on weekends was related to worse glycemic control in T2DM patients independently of sleep disturbances [30]. What’s more, a randomized control trial involving T2DM in those who slept after midnight by Li et al. showed that intervention by combined education (diabetes and sleep education) could greatly benefit glycemic control and decrease the degree of insulin resistance [31]. In addition, consistent with other findings [32], long night sleep duration increased the risk of T2DM.

However, associations between the midpoint of sleep and T2DM were inconsistent with previous studies. This study showed that early midpoint of sleep also increased the T2DM risk. A cross-sectional study involving 821 participants reported that morningness was associated with diabetes in middle-aged and elderly people [33]. One possible explanation for the differences observed between these studies is that there are large differences in circadian patterns between rural and urban populations [34]. The rural population presented a more predominantly early sleep pattern, as determined by the mid-sleep phase. In the present study, people might have an early midpoint of sleep due to work schedule, suggesting that a mismatch between internal circadian rhythms and social factors could play a significant role in the development of diabetes [35]. Though there were no objective parameters of chronotype in the present study, the association between chronotype and rural population should be addressed in future studies.

Two previous studies were conducted to investigate the combined effects of sleep behaviors and T2DM. Liu et al. showed that long napping and night sleep duration might increase the risk of T2DM combinedly [6]. Lou et al. presented that short sleep duration and worse sleep quality might be jointly related to higher odds of T2DM [36]. The present study found that midpoint of sleep and long night sleep duration might have combined associations with the prevalence of T2DM. Investigating combined associations of midpoint of sleep and night sleep duration simultaneously with T2DM might have important implications for understanding the impact of sleep on T2DM.

Gender and age can modify the detrimental effects of the midpoint of sleep on T2DM. Consistent with our results, Fabbian et al. reported that eveningness may impact general health especially in women [11]. However, in one analysis of 1620 middle-aged Korean men and women (age range 47–59 years), stronger associations of Evening Chronotype with T2DM were presented among men [12]. Consistent with Liu et al. [12] that women were more vulnerable to the adverse effects of night sleep duration, we also found that short and long night sleep duration had more effects on women. Different physiological and lifestyles may contribute to the gender differences. Individuals with younger age tend to under a mismatch between circadian and social activities such as working and studying and have adverse health outcomes [35, 37].

All these above findings supported that disruption of the circadian clock was associated with adverse changes in T2DM. The biological mechanisms underlying the relationship of the midpoint of sleep and night sleep duration with T2DM were not fully clear. One possible explanation is that circadian misalignment leads to metabolism and endocrine alterations. Reduced glucose tolerance and thyrotropin concentrations, elevated evening cortisol concentrations and sympathetic nervous system activity may facilitate the onset of T2DM [38]. Previous experimental studies had proved the adverse cardiovascular metabolism outcomes of circadian misalignment both in humans [39–41] and in animals [42–44]. Another is that nocturnal melatonin, a hormone that may be related to insulin-sensitive circadian rhythm regulation, may be disturbed by sleep deprivation, circadian rhythm disorders and/or light exposure [45]. Additionally, circadian rhythm was associated with dietary intake and dietary behavior. An animal experiment showed that the circadian rhythm of eating behavior of mice with clock gene mutations is weakened, which leads to glucose and lipid metabolism disorders [46].

Several limitations are needed to address. First, the midpoint of sleep was calculated using sleep time and wake-up time of the previous month, but the midpoint of sleep was different between free days and work days and it is reported that free day midpoint of sleep was correlated better with chronotype scores assessed by chronotype questionnaire. However, because of the hot weather and any other reason (i.e. farming), most of the participants were on vacation during baseline assessment. Therefore, the participants probably had free time during the past month. Second, the study did not consider non-holiday seasons, where work is a major factor in sleep deprivation. Due to work, people may have late daily schedule and larger social jetlag, which may increase the risk of the prevalenceT2DM. The association between sleep and T2DM may be underestimated in the present study. Third, there were no objective parameters of chronotype in the present study which should be addressed in future studies. Fourth, the midpoint of sleep was classified into three categories by the 25th and 75th percentile based on a previous study [26] and might lead to misclassification. Fifth, the information about sleep disorders was not controlled in the present study. Some of the sleep outcomes might be due to the presence of sleep disorder (sleep apnea). It is better to use more accurate ways such as polysomnography to evaluate sleep in future studies. Lastly, this study was cross-sectional that cannot fully demonstrate a clear cause-effect conclusion between midpoint of sleep or night sleep duration and T2DM. Self-reported sleep information might also cause recall bias.

## Conclusion

In conclusion, Late and early midpoint of sleep and long night sleep duration were all related to higher odds of T2DM. Meanwhile, the midpoint of sleep and long night sleep duration might jointly increase the prevalence of T2DM. Moreover, the association was more obvious in women. These findings indicate that circadian rhythm is a crucial factor associated with adverse health outcomes in rural populations, especially among rural women. Given the importance of circadian rhythm in metabolism, further studies regarding the potential role of chronotype in diabetes prevention should be explored.

## Supplementary Information

**Additional file 1: Supplementary Table 1.** The demographic characteristics of subjects according to midpoint of sleep. **Supplementary Table 2.** Comparison of bedtime, wake-up time and sleep duration across 3 categories of the midpoint of sleep. **Supplementary Table 3.** Subgroup analysis of the association of midpoint of sleep and night sleep duration with T2DM.

## Data Availability

The datasets used and/or analyzed during the current study are available from the corresponding author on reasonable request.

## Abbreviations

BMI: Body mass index
CI: Confidence interval
T2DM: Type 2 diabetes mellitus
SD: Standard deviation
