# Supplementary material for: Associations of midpoint of sleep and night sleep duration with type 2 diabetes mellitus in Chinese rural population: the Henan rural cohort study
Source: BMC Public Health. 2021 May 7;21:879. doi: 10.1186/s12889-021-10833-6 (PMC8106181; doi:10.1186/s12889-021-10833-6)
Supplement: Supplementary file 1 — Additional file 1: Supplementary Table 1. The demographic characteristics of subjects according to midpoint of sleep. Supplementary Table 2. Comparison of bedtime, wake-up time and sleep duration across 3 categories of the midpoint of sleep. Supplementary Table 3. Subgroup analysis of the association of midpoint of sleep and night sleep duration with T2DM. [file 12889_2021_10833_MOESM1_ESM.docx]

**Supplementary Table 1. The demographic characteristics of subjects according to midpoint of sleep**

| **Variables** | **Early** | **Intermediate** | **Late** | ***P*** |
| --- | --- | --- | --- | --- |
| N | 11560 | 16456 | 9260 |  |
| Age(year), mean±SD | 61.02±9.72 | 56.03±11.08 | 49.66±13.34 | <0.001 |
| Gender, n (%) |  |  |  | <0.001 |
| Men | 4664(40.35) | 6220(37.80) | 3572(38.57) |  |
| Women | 6896(59.65) | 10236(62.20) | 5688(61.43) |  |
| Marital status, n (%) |  |  |  | <0.001 |
| Married/cohabitation | 10032(86.78) | 14974(90.99) | 8368(90.37) |  |
| Unmarried/divorced/widowed | 1528(13.22) | 1428(9.01) | 892(9.63) |  |
| Educational levels, n (%) |  |  |  | <0.001 |
| Primary school or below | 6970(60.29) | 7243(44.01) | 2929(31.63) |  |
| Junior high school | 3712(32.11) | 6929(42.11) | 4018(43.39) |  |
| Senior high school or above | 878(7.60) | 2284(13.88) | 2313(24.98) |  |
| Average income per month, n (%) |  |  |  | <0.001 |
| <500 RMB | 4951(42.83) | 5877(35.71) | 2727(29.45) |  |
| 500- RMB | 3643(31.51) | 5518(33.53) | 3140(33.91) |  |
| ≥1000 RMB | 2966(25.66) | 5061(30.75) | 3393(36.64) |  |
| High vegetables and fruits intake, n (%) | 4529(39.18) | 7246(44.04) | 3859(41.68) | <0.001 |
| High fat diet, n (%) | 1861(16.10) | 3020(18.35) | 2174(23.48) | <0.001 |
| Current smoking, n (%) | 1879(16.25) | 2888(17.55) | 2160(23.33) | <0.001 |
| Current drinking, n (%) | 1737(15.03) | 2822(17.15) | 1988(21.47) | <0.001 |
| Physical activity, n (%) |  |  |  | <0.001 |
| Light | 3551(30.72) | 4985(30.29) | 3423(36.97) |  |
| Moderate | 4178(36.14) | 6226(37.83) | 3680(39.74) |  |
| Vigorous | 3831(33.14) | 5245(31.87) | 2157(23.29) |  |
| Family history of diabetes, n (%) | 310(2.68) | 728(4.42) | 489(5.28) | <0.001 |
| Body mass index, mean±SD | 24.64±3.57 | 24.91±3.52 | 24.88±3.64 | <0.001 |
| Night sleep duration, mean±SD | 8.19±1.24 | 7.72±1.09 | 7.32±1.45 | <0.001 |
| Napping duration, mean±SD | 58.36±49.75 | 57.99±49.62 | 55.31±52.48 | <0.001 |
| T2DM, n (%) | 1324(11.45) | 1483(9.01) | 773(8.35) | <0.001 |

**Supplementary Table 2. Comparison of bedtime, wake-up time and sleep duration across 3 categories of the midpoint of sleep**

| **Characteristic** |  | **Total** | | | |  | **Men** | | | |  | **Women** | | | |
| --- | --- | --- | --- | --- | --- | --- | --- | --- | --- | --- | --- | --- | --- | --- | --- |
|  |  | Early | Intermediate | Late | P |  | Early | Intermediate | Late | P |  | Early | Intermediate | Late | P |
| Bedtime |  | 20:46 | 21:46 | 22:43 | <0.001 |  | 20:46 | 21:48 | 22:55 | <0.001 |  | 20:47 | 21:44 | 22:35 | <0.001 |
|  |  | ±47 min | ±38 min | ±56 min |  |  | ±47 min | ±38 min | ±58 min |  |  | ±48 min | ±38 min | ±55 min |  |
| Wake-up time |  | 5:11 | 5:47 | 6:36 | <0.001 |  | 5:09 | 5:46 | 6:41 | <0.001 |  | 5:13 | 5:49 | 6:34 | <0.001 |
|  |  | ±41 min | ±34 min | ±53 min |  |  | ±41 min | ±35 min | ±56 min |  |  | ±40 min | ±34 min | ±51 min |  |
| Night sleep duration |  | 8h11 | 7h43 | 7h19 | <0.001 |  | 8h11 | 7h41 | 7h19 | <0.001 |  | 8h12 | 7h44 | 7h19 | <0.001 |
|  |  | ±74 min | ±65 min | ±87 min |  |  | ±74 min | ±67 min | ±85 min |  |  | ±74 min | ±64 min | ±89 min |  |

**Supplementary Table 3. Subgroup analysis of the association of midpoint of sleep and night sleep duration with T2DM**

| Category | **OR (95%CI)** | | | |  | **OR (95%CI)** | | | |
| --- | --- | --- | --- | --- | --- | --- | --- | --- | --- |
|  | Early | Intermediate | Late | P for interaction |  | <5 | 7- | ≥10 | P for interaction |
| Age |  |  |  | <0.001 |  |  |  |  | 0.117 |
| <60 | 1.32(1.15-1.52) | 1.00 | 0.92(0.80-1.05) |  |  | 1.17(0.74-1.86) | 1.00 | 1.25(0.91-1.73) |  |
| ≥60 | 1.11(1.00-1.23) | 1.00 | 1.20(1.04-1.39) |  |  | 1.09(0.75-1.59) | 1.00 | 1.33(1.09-1.62) |  |
| BMI |  |  |  | 0.375 |  |  |  |  | 0.100 |
| <24 | 1.10(0.95-1.28) | 1.00 | 1.03(0.85-1.25) |  |  | 1.07(0.63-1.81) | 1.00 | 1.36(1.04-1.80) |  |
| ≥24 | 1.11(1.00-1.22) | 1.00 | 1.18(1.05-1.33) |  |  | 1.21(0.86-1.70) | 1.00 | 1.20(0.98-1.48) |  |
| Family history of diabetes |  |  |  | 0.142 |  |  |  |  | 0.331 |
| No | 1.16(1.07-1.27) | 1.00 | 1.16(1.04-1.29) |  |  | 1.12(0.83-1.52) | 1.00 | 1.26(1.06-1.49) |  |
| Yes | 0.73(0.51-1.03) | 1.00 | 0.98(0.70-1.37) |  |  | 1.19(0.42-3.38) | 1.00 | 1.69(0.86-3.31) |  |
| Physical activity |  |  |  | 0.428 |  |  |  |  | 0.548 |
| Low | 1.17(1.02-1.34) | 1.00 | 1.10(0.94-1.29) |  |  | 0.88(0.52-1.48) | 1.00 | 1.37(1.07-1.74) |  |
| Moderate | 1.12(0.98-1.29) | 1.00 | 1.11(0.94-1.31) |  |  | 1.47(0.94-2.28) | 1.00 | 1.24(0.91-1.69) |  |
| High | 1.06(0.90-1.24) | 1.00 | 1.25(1.02-1.54) |  |  | 0.97(0.54-1.73) | 1.00 | 1.22(0.85-1.74) |  |
| Smoking |  |  |  | 0.041 |  |  |  |  | 0.399 |
| Never | 1.11(1.01-1.22) | 1.00 | 1.09(0.97-1.23) |  |  | 1.30(0.94-1.80) | 1.00 | 1.25(1.04-1.51) |  |
| Former | 1.07(0.83-1.38) | 1.00 | 1.00(0.71-1.41) |  |  | 0.67(0.13-3.43) | 1.00 | 1.47(0.91-2.37) |  |
| Current | 1.19(0.94-1.51) | 1.00 | 1.32(1.04-1.67) |  |  | 0.74(0.35-1.54) | 1.00 | 1.22(0.72-2.06) |  |
| Drinking |  |  |  | 0.043 |  |  |  |  | 0.088 |
| Never | 1.11(1.01-1.21) | 1.00 | 1.08(0.97-1.22) |  |  | 1.35(0.98-1.86) | 1.00 | 1.27(1.06-1.53) |  |
| Former | 1.58(1.12-2.24) | 1.00 | 1.60(1.05-2.44) |  |  | 1.49(0.46-4.86) | 1.00 | 1.24(0.64-2.41) |  |
| Current | 1.06(0.84-1.33) | 1.00 | 1.22(0.96-1.54) |  |  | 0.46(0.19-1.13) | 1.00 | 1.34(0.84-2.14) |  |

Adjusted for age, gender, marital status, educational levels, average income per month, high vegetables and fruits intake, high fatty diet, smoking status, drinking status, physical activity, body mass index, family history of diabetes, napping duration, sleep quality, snoring, night sleep duration or midpoint of sleep
